# Development of On-Chip Multi-Imaging Flow Cytometry for Identification of Imaging Biomarkers of Clustered Circulating Tumor Cells

**DOI:** 10.1371/journal.pone.0104372

**Published:** 2014-08-20

**Authors:** Hyonchol Kim, Hideyuki Terazono, Yoshiyasu Nakamura, Kazuko Sakai, Akihiro Hattori, Masao Odaka, Mathias Girault, Tokuzo Arao, Kazuto Nishio, Yohei Miyagi, Kenji Yasuda

**Affiliations:** 1 Kanagawa Academy of Science and Technology, Takatsu, Kawasaki, Japan; 2 Department of Biomedical Information, Division of Biosystems, Institute of Biomaterials and Bioengineering, Tokyo Medical and Dental University, Chiyoda, Tokyo, Japan; 3 Molecular Pathology and Genetics Division, Kanagawa Cancer Center Research Institute, Asahi-ku, Yokohama, Japan; 4 Department of Genome Biology, School of Medicine, Kinki University, Osaka-Sayama, Osaka, Japan; Witten/Herdecke University, Germany

## Abstract

An on-chip multi-imaging flow cytometry system has been developed to obtain morphometric parameters of cell clusters such as cell number, perimeter, total cross-sectional area, number of nuclei and size of clusters as “imaging biomarkers”, with simultaneous acquisition and analysis of both bright-field (BF) and fluorescent (FL) images at 200 frames per second (fps); by using this system, we examined the effectiveness of using imaging biomarkers for the identification of clustered circulating tumor cells (CTCs). Sample blood of rats in which a prostate cancer cell line (MAT-LyLu) had been pre-implanted was applied to a microchannel on a disposable microchip after staining the nuclei using fluorescent dye for their visualization, and the acquired images were measured and compared with those of healthy rats. In terms of the results, clustered cells having (1) cell area larger than 200 µm^2^ and (2) nucleus area larger than 90 µm^2^ were specifically observed in cancer cell-implanted blood, but were not observed in healthy rats. In addition, (3) clusters having more than 3 nuclei were specific for cancer-implanted blood and (4) a ratio between the actual perimeter and the perimeter calculated from the obtained area, which reflects a shape distorted from ideal roundness, of less than 0.90 was specific for all clusters having more than 3 nuclei and was also specific for cancer-implanted blood. The collected clusters larger than 300 µm^2^ were examined by quantitative gene copy number assay, and were identified as being CTCs. These results indicate the usefulness of the imaging biomarkers for characterizing clusters, and all of the four examined imaging biomarkers—cluster area, nuclei area, nuclei number, and ratio of perimeter—can identify clustered CTCs in blood with the same level of preciseness using multi-imaging cytometry.

## Introduction

Finding irregular cells in blood is fundamental to achieving non-invasive health checks, such as cancer and immune diagnostics. For example, circulating tumor cells (CTCs) are expected to form additional seeds for subsequent growth of tumors [1]–[3], and quantitative detection of CTCs in the blood [4]–[8] has the potential to achieve minimally invasive cancer diagnosis in comparison with conventional biopsies. One major approach to finding irregular cells is the targeting of specific molecules, molecular biomarkers, on the cell surface [1], [3], [6], [9], [10]; however, its application has sometimes had the difficulty of false-negative detection because of the variety of molecular expression properties of targeted cells.

To overcome these difficulties, we developed another system for the recognition of target cells [11]–[13]. In this system, cell samples were applied to a microchannel fabricated on a small microchip, cellular images were taken with a high-speed CCD camera, and target cells were identified depending on their morphological characteristics, such as cellular area and perimeter. These morphological parameters, referred to as “imaging biomarkers” hereafter, are other indexes to identify specific target cells. For example, a large cellular size was indicated for some tumor cells [14]–[17], and a larger nucleus than in healthy cells is known as one common property of the morphometric phenotype of cancer cells [18]–[24]; therefore, finding target cells using imaging biomarkers, especially using both cell size and nucleus conformation, is useful for the identification of tumor cells. In this study, a real-time cell sorting system to achieve simultaneous processing of imaging biomarkers for both optical image (i.e., total cell configuration) and fluorescent image (i.e., nucleus configuration) was developed, and it was applied to identify irregular cells, especially clustered cells, in a blood sample. According to previous reports on CTC detection, the possibility of the CTCs forming clusters was suggested [7]; however, clear evidence had not been identified and there have been no quantitative studies on the identification of clustered cells in the blood. Here, a quantitative approach for cluster detection was suggested using imaging biomarkers as detection indexes.

## Materials and Methods

### Fabrication of microchip

The microchip was fabricated by the following procedure. A mask blank, which was a glass substrate coated with both chromium for light interception and positive photo-resist (AZP1350) for the fabrication of patterns (CBL4006Du-AZP, Clean Surface Technology Co., Kanagawa, Japan), was set to a laser lithography system (DDB-3TH, Neoark, Co., Tokyo, Japan) and a laser (405 nm wavelength) was irradiated onto the mask blank in the same pattern as the microchannel used in this study. After the irradiation, the mask blank was immersed in a developer of the resist (NMD-3, Tokyo Ohka Kogyo Co., Kanagawa, Japan) to remove the resist on which the laser was irradiated; then, a chromium layer was bared at this position. Next, the mask blank was immersed in chromium etching solution (MPM-E350, DNP Fine Chemicals, Co., Kanagawa, Japan), after which the bared chromium layer was removed and a transparent pattern of the microchannel was formed on the substrate. Finally, the whole resist on the mask blank was removed by light irradiation onto the whole of the substrate and immersion of the substrate in the developer; then, a photo mask of the microchannel was fabricated.

On the other hand, a light-curing resin (SU-8 3025, Nippon Kayaku Co., Tokyo, Japan) was spin-coated using a spin coater (1H-DX2, Mikasa, Co., Tokyo, Japan) of 25 µm thickness on a clean Si substrate. The resin-coated substrate was pre-baked at 95°C for 15 min, set in a mask aligner with the fabricated photo mask (MA-20, Mikasa), and the light (365 nm wavelength) was irradiated through the mask to harden the resin with the pattern of the microchannel. The substrate was heated at 65°C for 1 min and 95°C for 5 min sequentially to promote hardening of the resin, and excess resin was removed by immersing the substrate in SU-8 developer (Nippon Kayaku). A mold of the microchannel was then fabricated using the resin on the Si substrate.

To fabricate the chip, poly(dimethylsiloxane) (PDMS; SYLGARD 184 silicon elastomer, Dow Corning Co., Midland, MI, USA) was dropped onto the fabricated mold in sol state, and heated at 90°C for 1 h to harden the PDMS. The PDMS on which the pattern of the microchannel was transferred was peeled off from the mold and stuck with cleaned cover glass. Finally, plastic columns for the application of solvents including sample blood were pasted on the PDMS with epoxy resin; then, the microchip to be used in this study was fabricated.

### Preparation of sample blood

This study was carried out in strict accordance with the Act on Welfare and Management of Animals of the Ministry of the Environment, Japan. The protocol was approved by the animal experiment committee of the Kanagawa Cancer Center (permit number 21-02). MAT-LyLu is a rat prostate cancer cell line established from the original Dunning R3327 tumor maintained by *in vivo* passage of a prostate cancer that spontaneously occurred in a Copenhagen rat [25]. This cell line was a generous gift from the original founders through Hisao Ekimoto, Ph.D., at the Oncology Section, Laboratory of Biology, Nippon Kayaku Co., Ltd., and was maintained in our laboratory.

To obtain blood containing cancer cells, the MAT-LyLu was adjusted to 5×10^6^ cells in 200 µL of cell culture medium (RPMI 1640, Life Technologies Co., Grand Island, NY, USA), and implanted into the dorsal subcutaneous tissue of a Copenhagen rat (male, 6 weeks old). At 2 weeks after implantation, blood of the rat was collected from the subclavian vein using a collection tube containing heparin. The blood was hemolyzed using commercial reagent (BD Pharm Lyse, without fixative, BD Biosciences, San Jose, CA, USA) for 10 min, washed along with 200× g centrifugation for 5 min and re-suspended two times in phosphate-buffered saline (PBS) containing 1% bovine serum albumin, suspended in PBS containing 100 ng/mL Hoechst 33258, and then incubated for 10 min to stain cellular nuclei. The sample was washed again along with centrifugation 3 times, suspended in 5% glucose solution, and applied to the sample inlet on the chip.

### Flow cytometry

The prepared sample blood was applied to the sample inlet on a fabricated microchip with a sample volume of 50 µL in an assay. The same buffer with the sample cell suspension (i.e., 5% glucose) was also used as a sheath buffer, and was applied to the sheath buffer inlet. Air pressure was applied onto both sample and sheath buffer inlets simultaneously using a syringe pump to introduce these liquids into the microchannels. Before starting the experiments, flow velocity was calibrated by taking images of calibration beads using a CCD camera (Ditect Co., Tokyo, Japan) as the shift of bead position in the microchannel within a few frames of the images, and typically, 1 kPa pressure achieved flow velocity of about 3 mm/sec at the position after the meeting of sample and sheath flows. Multi-imaging observations of sample blood were then performed through the multi-view unit with 3 mm/sec flow velocity and 200 fps acquisition rate.

### Comparative genomic hybridization analysis

Rat genome comparative genomic hybridization (CGH) microarray 244A (Agilent Technologies, Santa Clara, CA, USA) was used to perform array CGH on genomic DNA obtained from the MAT-LyLu cell line according to the manufacturer's instructions. A DNA sample obtained from liver tissue of a healthy Copenhagen rat was used as a reference. Genomic DNAs were extracted using a QIAamp DNA Mini kit (Qiagen, Hilden, Germany) according to the manufacturer's instructions. The DNA concentration was determined with PicoGreen dsDNA Quantitation Reagent (Life Technologies). Agilent Genomic Workbench (Agilent Technologies) was used to analyze chromosomal patterns using an ADM-2 algorithm setting a threshold of 5.0.

### Copy number assay

The gene copy numbers for *csrp2* and *zdhhc17* were determined using TaqMan Copy Number Assays according to the manufacturer's instructions (Applied Biosystems, Foster City, CA, USA). The gene-specific primers and TaqMan probes were used in the experiments had the following sequences. Rat *csrp2* primers were: sense, 5′-GGACTAAATGGATTGATGCCACTCT-3′; antisense, 5′-GTCCCTGCTTCAAAGAACTGTCT-3′; probe, 5′-FAM-AAGAGCAAGAAAGGAAACCC-MGB-NFQ-3′. Rat *zdhhc17* primers were: sense, 5′-GCCCTACTGCATGCATGATACA-3′; antisense, 5′-GGGCTGTTTTGCACATGAAATTCAA-3′; probe, 5′-FAM-CTGGACAGCATCTGCTAGTATAC-MGB-NFQ-3′. Rat *rpp40* primers were: sense, 5′-GTATGACACTGGCATGGAAGTCT-3′; antisense, 5′-CTTGCAGGTCCTCTGTGGAT-3′; probe, 5′-FAM-CCTGGCAATCAAAGTTAGGCTTAG-MGB-NFQ-3′. Genomic DNAs obtained from collected samples using the cell sorting system were extracted using a QIAamp DNA Micro kit (Qiagen) according to the manufacturer's instructions. The DNA concentrations were determined with PicoGreen dsDNA Quantitation Reagent. Rat *rpp40* was used as an internal control. Genomic DNAs obtained from MAT-LyLu cell line and healthy rat liver were used as positive and negative controls, respectively.

## Results

### Development of on-chip multi-imaging flow cytometry system

**Figure 1:**
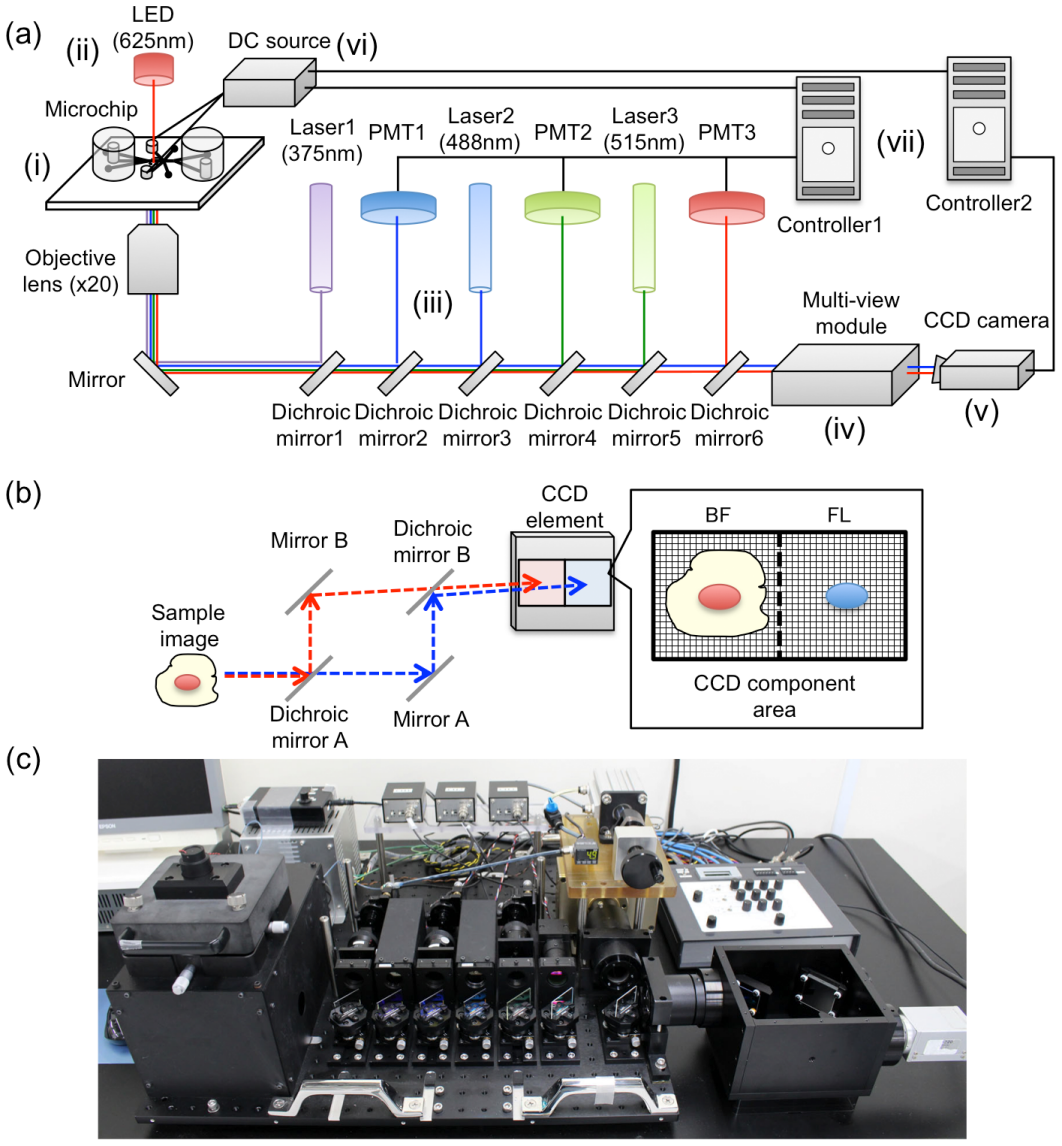
Instrumental set-up. (a) Summary of the on-chip multi-imaging flow cytometry system. The system was composed of seven major modules: (i) microchip, (ii) bright-field (BF) imaging, (iii) fluorescent (FL) detection, (iv) multi-view, (v) CCD camera, (vi) sorting, and (vii) controller, as numbered in the figure. (b) Summary of the multi-view module. (c) A photograph of the system.

The on-chip multi-imaging flow cytometry system (Fig. 1) was composed of seven major modules as an improvement of previous systems [11]–[13], [26]: (i) microchip, (ii) bright-field (BF) light source, (iii) fluorescent (FL) excitation and detection, (iv) multi-view, (v) CCD camera, (vi) sorting, and (vii) controller, as numbered in Fig. 1 (a). In the BF light source module, an LED (625 nm wavelength) was used as a source for taking BF images and was irradiated from the top of the chip. This allowed simultaneous measurements of both BF and FL images, avoiding interference of the wavelengths during the measurements. An objective lens having 20× magnification and a 0.75 numerical aperture was set to the system, which allowed clear cell images to be taken within the depth range of the microchannel (25 µm) [27]. The FL excitation and detection modules contained three excitation lasers (375, 488, and 515 nm) and photomultipliers (PMTs), respectively, to monitor three different FL signals, which allowed conventional FL detection with labeling of target biomarkers. The controller module consisted of two independent units: one calculated FL signals and the other processed imaging biomarkers in multi-view images. Maximum frequencies of calculations were 10,000 frequencies per second (fps) for controller 1, which calculated FL intensities, and 200 fps for controller 2, which processed imaging biomarkers for the current system. According to the adjustment of suitable thresholds for these parameters, feedback signals could be sent to the sorting module. The sorting module was composed of a direct current (DC) source and electrodes connected with a microchip, and could apply DC voltages to cells flowing in a microchannel of the chip to purify target cells under feedback signals, if necessary. Figure 1 (b) shows the principle of the multi-view module [27], [28] used in this study. Firstly, optical paths between BF (red) and FL (blue) lights were separated using dichroic mirror A, as indicated in the figure. Next, angles of mirrors A and B were adjusted; then, BF and FL images were projected onto each half of a CCD component in the camera. An overview of the total system is shown in Fig. 1 (c). The system has a desktop size of 60 cm×60 cm.

**Figure 2:**
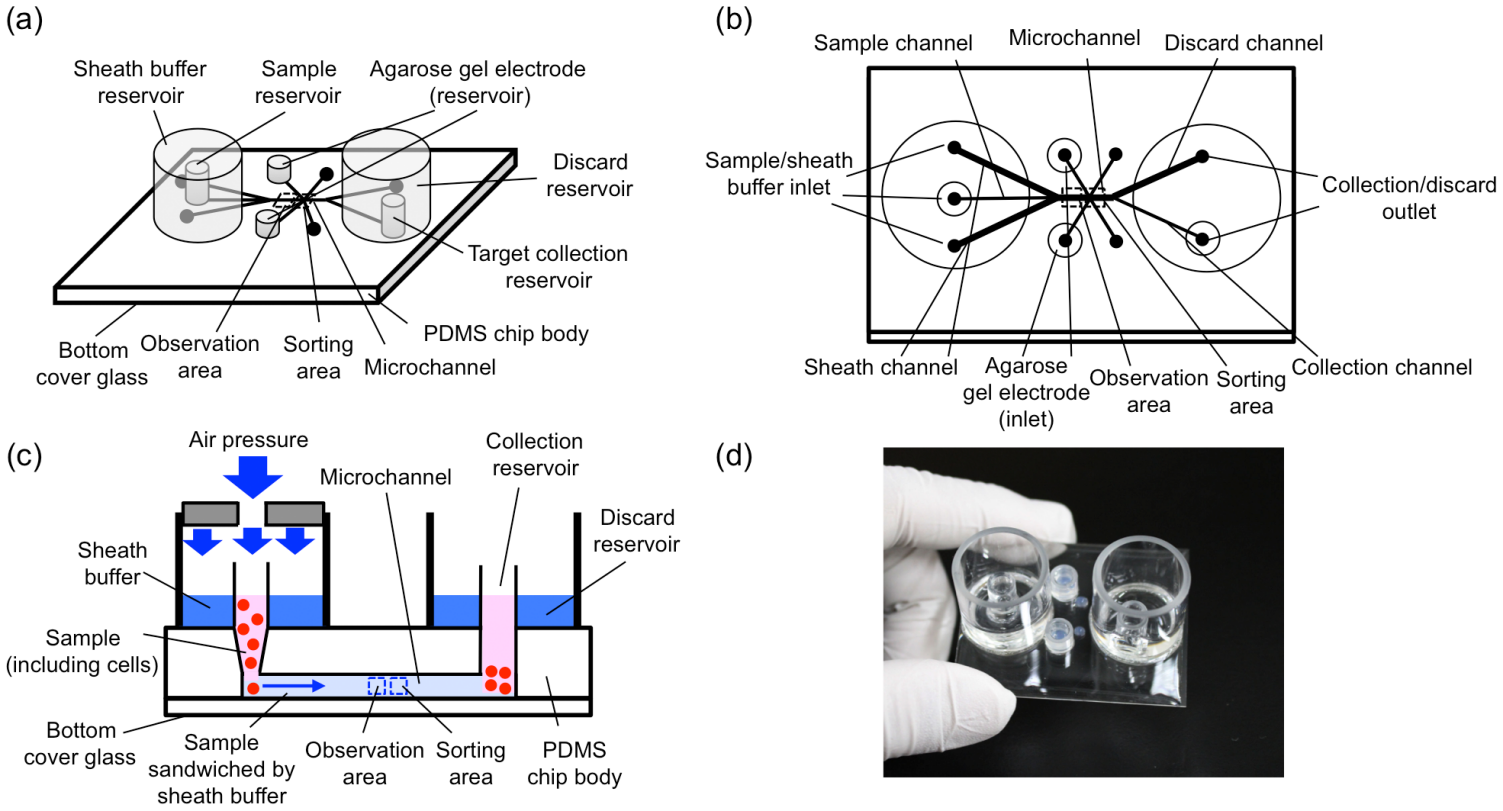
Overview of the microchip. (a) Diagonal, (b) top, and (c) side views of the microchip used in this study. (d) A photograph of the chip. Total chip size is 50 mm×40 mm.

Figure 2 shows the microchip designed to be suitable for this study. The chip body was fabricated with poly(dimethylsiloxane) (PDMS) attached to a cover glass to apply optical transparency in the observation. Microchannels were placed between the PDMS and the bottom cover glass in the chip with a 2 mmφ buffer entrance penetrating the PDMS. The upper stream of the microchannel was branched into three channels: the center connected with the sample inlet and the others were a sheath buffer inlet. Both sample and sheath buffers were introduced into the channel with application of air pressure onto both sample and sheath buffer inlets, simultaneously (Fig. 2 (c)). After the meeting of sample and sheath flows, the width of the sample flow was focused in the central one-third, which allowed imaging of each single cell upon the arrangement of all the cells in a straight line.

**Figure 3:**
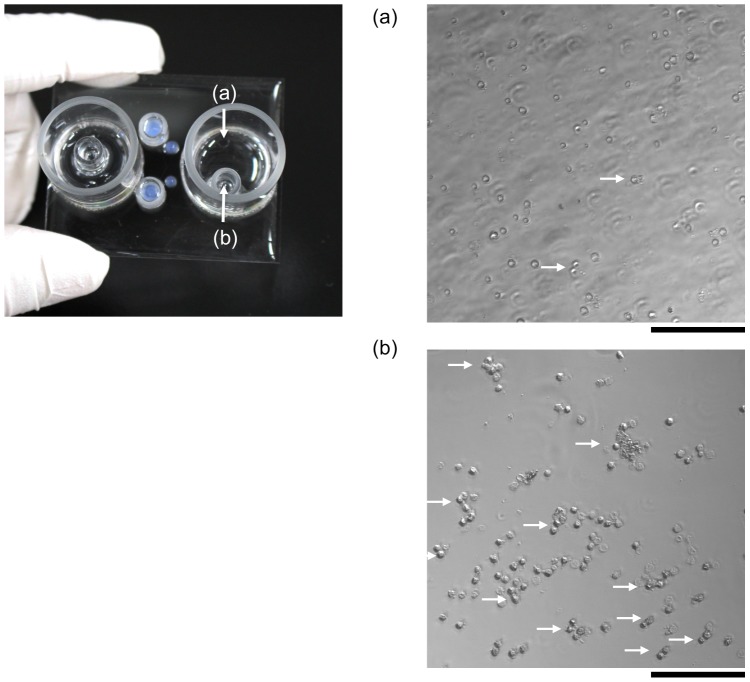
An example of cell sorting. Two photographs of the discarded reservoir (a) and the collection reservoir (b) indicated in the chip photograph are shown. Clustered cells are indicated by white arrows. Bars, 100 µm.

Images of the linearly arranged cells were obtained through the multi-view module and processed by the system (see Fig. 1), and when a target cell was found, DC voltage (typically 40 V with 100 µsec length) was applied to the cell through the agarose gel electrode (Fig. 2 (a) and (b)) to change its course in the collection channel [11], [13]. Figure 3 shows a typical example of the cell sorting with a blood sample of a cancer-implanted rat. As shown in this figure, target cells were set into cell clusters having a large BF area, and once the value of the BF area of the observed cell exceeded the pre-adjusted threshold value, 300 µm^2^ in this model case, a sorting voltage was applied to the cell and, finally, target cells were collected into the target collection reservoir. Figures 3 (a) and (b) show pictures taken for discarding (a) and collection (b) reservoirs, respectively. As shown in Fig. 3 (b), large cell clusters (indicated by arrows in the figures) were collected into the collection reservoir. On the other hand, single cells or small cell clusters were collected into the discarding reservoir (Fig. 3 (a)), indicating the success of target collection using one imaging biomarker, BF area, as a collection parameter. The sorting capacity, which has been determined as the ratio between the number of target cells automatically detected by the system and the actual number of cells in the collection reservoir, was 24%. The low capacity of target cell collection was caused by the higher threshold setting in both recognition and collection processes to prevent ‘false positive’ sample collection. When the commercially available microbeads were used as a model target in this system, sorting capacity increased to 91%.

**Figure 4:**
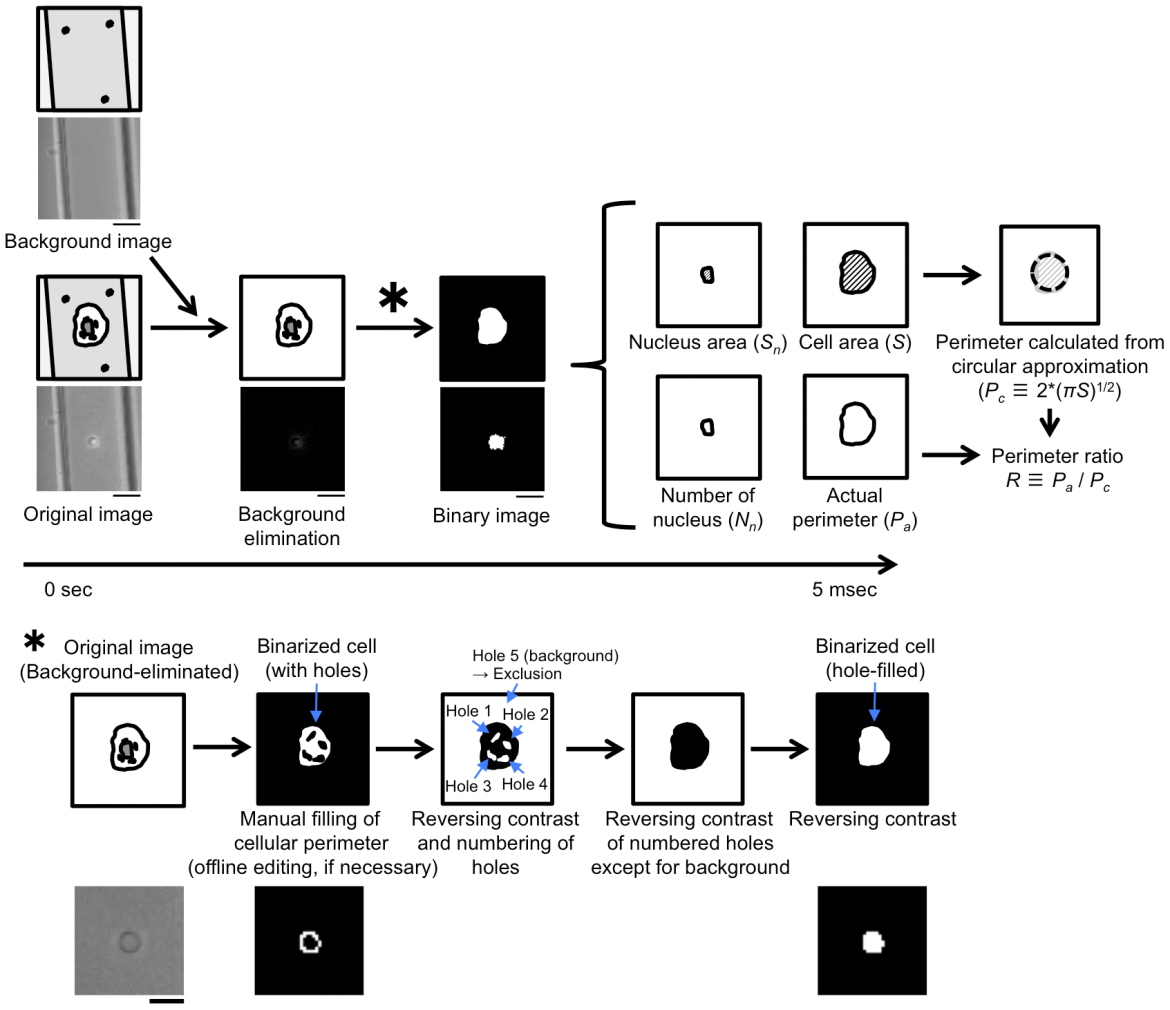
Summary of image processing. Firstly, photographs of both a cell and the background were taken. Next, the background image was subtracted from the cell image and holes were filled. Finally, imaging biomarkers, *S*, *S_n_*, *N_n_*, and *R*, were calculated. Bars, 10 µm. The hole-filling procedure is explained as indicated by an asterisk. Bar, 10 µm.

As shown in Fig. 3, target cells can be recognized by comparison of the imaging biomarkers with the threshold values pre-adjusted in the system. Figure 4 shows the detail of image processing in the system to obtain imaging biomarkers. Firstly, a background image, which was taken before the assay of flow cytometry, was subtracted from the obtained image with reductions of 8-bit grayscale values in each pixel. Next, the subtracted image was transformed to a binary image using a suitable threshold and pixel errors in the cell, which appeared by almost the same contrast in the cell as in the background, were filled (Fig. 4, asterisk); then, an extracted cell image was obtained. Finally, imaging biomarkers were calculated from the extracted cell image. In the current system, cell area (*S*) and actual perimeter (*P_a_*) were obtained from the BF image, and nucleus area (*S_n_*) and number of nuclei (*N_n_*) were obtained from the FL image. Additionally, the perimeter ratio, *R*, which was obtained as the ratio between *P_a_* and the perimeter calculated from *S* (*P_c_*) [29], was also obtained. These calculations were performed in real time at 200 fps using controller 2 in Fig. 1, and in this study, manual calculations of the imaging biomarkers, including a few modifications for apparently failed auto-calculations caused by the failure of continuous detection of the cell perimeter in the hole filling procedure, were also performed as post-processing to confirm the reliabilities of the obtained imaging biomarker values.

### Detection of clustered cells in cancer-implanted rat blood using imaging biomarkers

**Figure 5:**
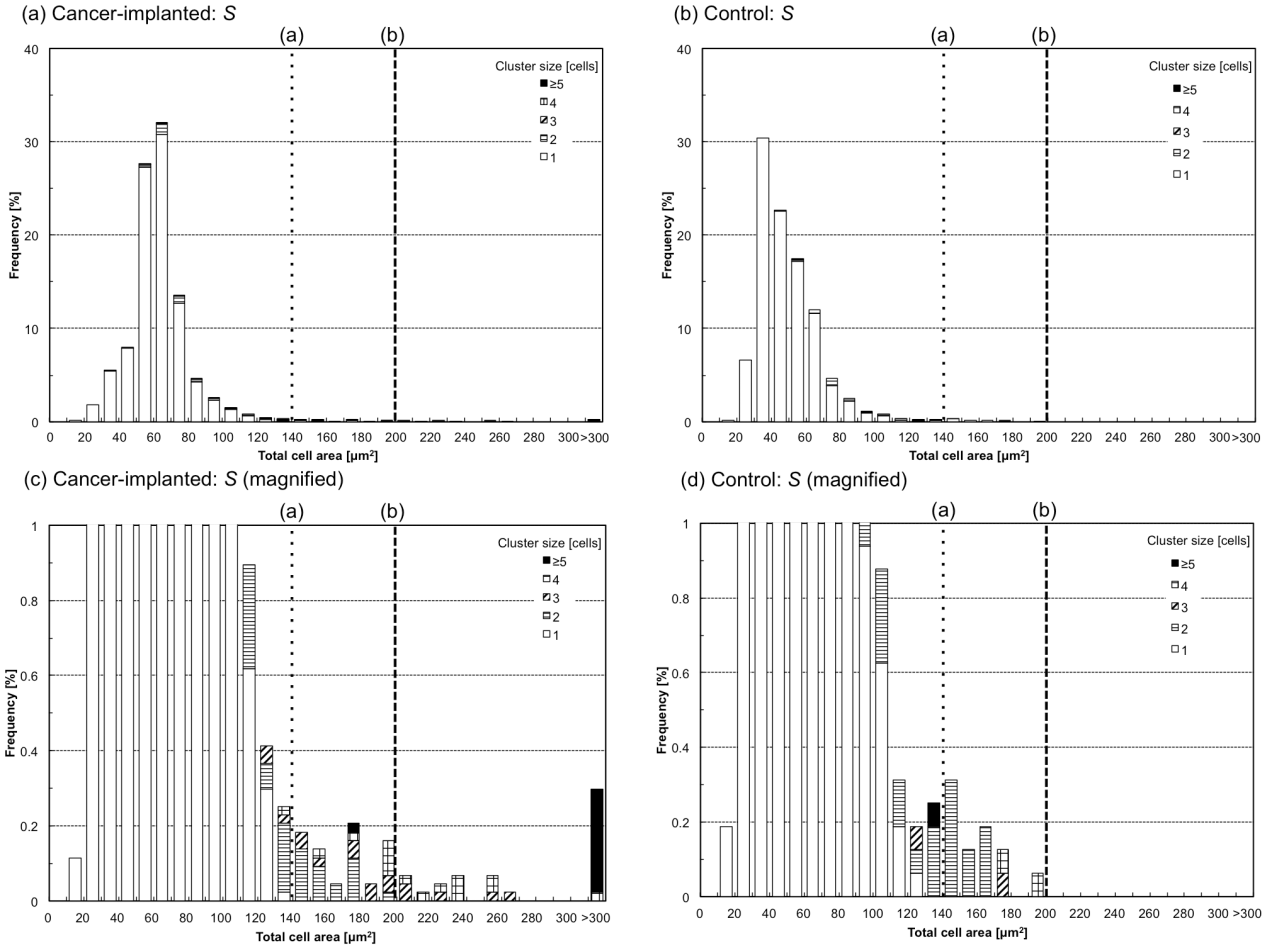
Histograms of total cell area, S, for cancer cell-implanted (a and c) and control blood (b and d). Two threshold values (a) and (b) for cluster identifications are indicated as dotted and dashed lines.

**Figure 6:**
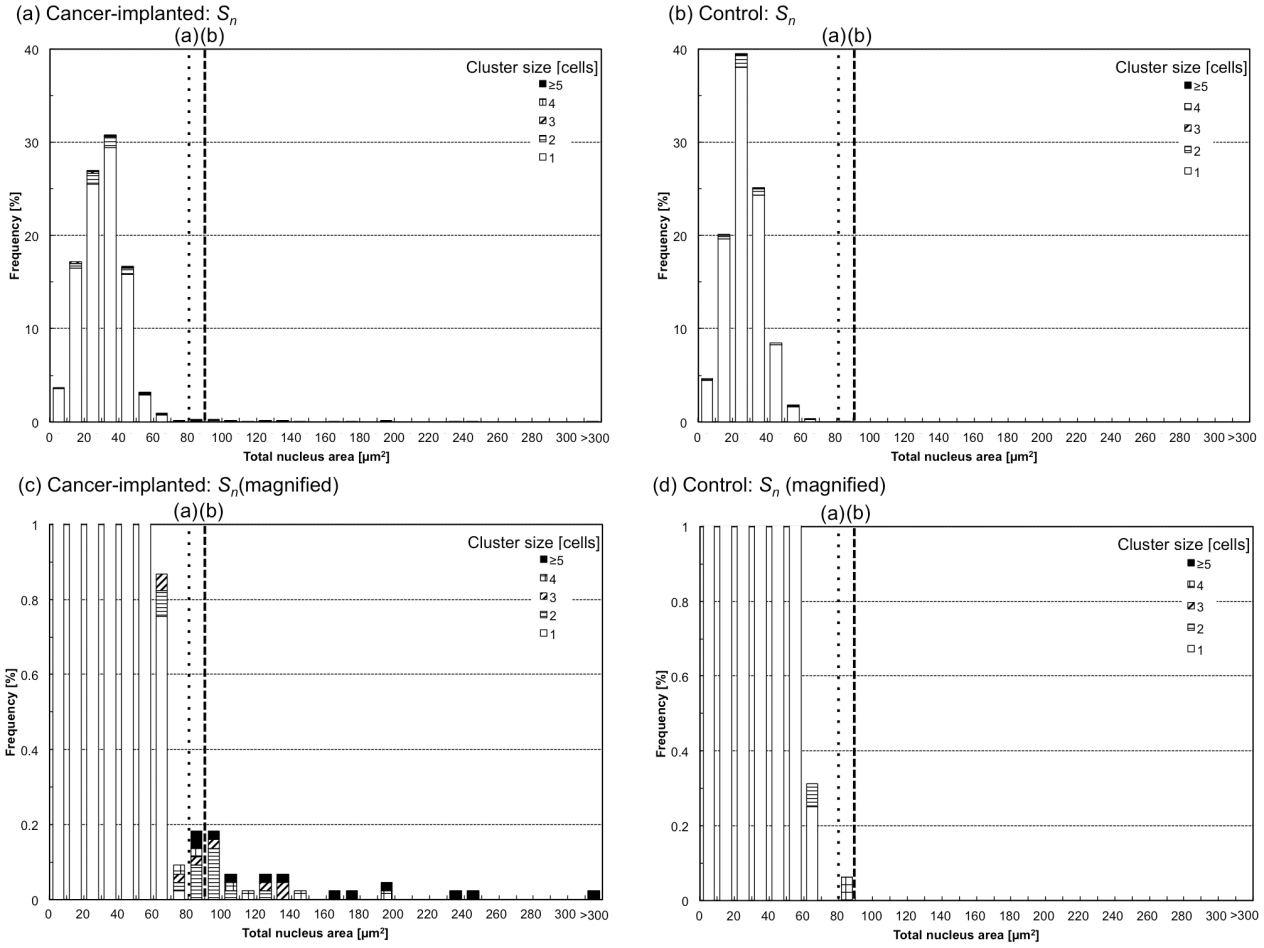
Histograms of total nucleus area, Sn, for cancer cell-implanted (a and c) and control blood (b and d). Two threshold values (a) and (b) for cluster identifications are indicated as dotted and dashed lines.

After the success of the system development, its performance for the identification of specific target cells using imaging biomarkers was quantitatively evaluated. Blood of a rat in which a rat prostate cancer cell line (MAT-LyLu) had been implanted was chosen as a model sample, and clustered cells in the blood were set as a target for the detection using imaging biomarkers with the developed system. One approach anticipated to achieve successful detection of the clusters is the use of cell area; therefore, areas in BF images (i.e., total cell area, *S*) and FL images (i.e., total nucleus area, *S_n_*) were measured using the system. Figures 5 and 6 are histograms of *S* (Fig. 5) and *S_n_* (Fig. 6) for cells in the cancer-implanted blood (N = 4375), shown with healthy rat blood as its control (N = 1599). Detailed numbers including *S* and *S_n_* are also summarized in Table 1. From the results, clustered cells were observed at a count of 237 in cancer-implanted samples (5.4% of the total) and a count of 56 in the control (3.5% of the total). In addition, two clear threshold values were found in both *S* and *S_n_*; that is, (a) all cells having *S* larger than 140 µm^2^ (count of 61, 1.4% of the total, for cancer-implanted samples and 13, 0.8% of the total, for the control) and *S_n_* larger than 80 µm^2^ (count of 34, 0.8% of the total, for cancer-implanted samples and 1, 0.1% of the total, for the control) were clustered cells, as indicated by the dotted lines in Figs. 5 and 6, and (b) the clustered cells having *S* larger than 200 µm^2^ (count of 27, 0.6% of the total) and *S_n_* larger than 90 µm^2^ (count of 26, 0.6% of the total) were specifically observed in cancer cell-implanted blood. These results indicate that some cell clusters can be identified by using *S* and *S_n_* (61 of 237, 26% of all clusters, for *S* and 34 of 237, 14% of all clusters, for *S_n_*) as parameters for detection.

**Table 1:** Summary of total cell area, *S*, total nucleus area, *S_n_*, number of nuclei, *N_n_*, and perimeter ratio, *R*, for each cluster size.

Cancer-implanted (N = 4375)			Total cell area, *S* [µm^2^]					Total nucleus area, *S_n_* [µm^2^]				
Cluster [cells]	Frequency [counts]	Frequency [%]	Average	Median	S.D.	Max.	Min.	Average	Median	S.D.	Max	Min
1	4138	94.58	62	62	15	133	11	30	31	12	73	7
2	174	3.98	88	75	33	194	36	36	33	19	122	10
3	33	0.75	133	124	58	263	53	45	33	35	134	12
4	16	0.37	219	208	57	393	136	74	52	46	195	24
≥5	14	0.32	515	421	180	1163	179	149	131	85	342	37
≥2	237	5.4	129	81	130	1163	36	47	35	41	342	10

Cancer-implanted (continued)			Number of nuclei, *N_n_*					Perimeter ratio, *R*				
Cluster [cells]	Frequency [counts]	Frequency [%]	Average	Median	S.D.	Max.	Min.	Average	Median	S.D.	Max	Min
1	4138	94.58	1.03	1.00	0.20	3	1	0.96	0.96	0.02	1.00	0.90
2	174	3.98	1.30	1.00	0.50	3	1	0.89	0.91	0.05	0.97	0.76
3	33	0.75	1.82	2.00	0.88	4	1	0.83	0.82	0.06	0.95	0.67
4	16	0.37	2.75	3.00	1.13	4	1	0.84	0.84	0.07	0.95	0.74
≥5	14	0.32	5.00	5.00	1.15	10	2	0.80	0.80	0.03	0.89	0.79
≥2	237	5.4	1.70	1.00	1.26	10	1	0.87	0.89	0.06	0.97	0.67

Control (N = 1599)			Total cell area, *S* [µm^2^]					Total nucleus area, *S_n_* [µm^2^]				
Cluster [cells]	Frequency [counts]	Frequency [%]	Average	Median	S.D.	Max.	Min.	Average	Median	S.D.	Max	Min
1	1543	96.50	48	44	16	121	11	27	26	10	69	7
2	49	3.06	99	83	36	169	40	28	28	12	61	7
3	3	0.19	119	127	62	177	54	23	24	10	32	13
4	2	0.13	187	187	14	197	177	51	51	43	81	20
≥5	2	0.13	116	116	24	133	99	32	44	18	57	32
≥2	56	3.5	104	88	39	197	40	29	28	14	81	7

Control (continued)			Number of nuclei, *N_n_*					Perimeter ratio, *R*				
Cluster [cells]	Frequency [counts]	Frequency [%]	Average	Median	S.D.	Max.	Min.	Average	Median	S.D.	Max	Min
1	1543	96.50	1.00	1.00	0.00	1	1	0.96	0.96	0.02	1.00	0.90
2	49	3.06	1.16	1.00	0.37	2	1	0.82	0.84	0.05	0.91	0.70
3	3	0.19	1.67	2.00	0.58	2	1	0.83	0.83	0.06	0.89	0.77
4	2	0.13	1.50	1.50	0.71	2	1	0.81	0.81	0.08	0.86	0.75
≥5	2	0.13	1.00	1.00	0.00	1	1	0.81	0.81	0.01	0.82	0.80
≥2	56	3.5	1.20	1.00	0.40	2	1	0.82	0.83	0.05	0.91	0.70

**Figure 7:**
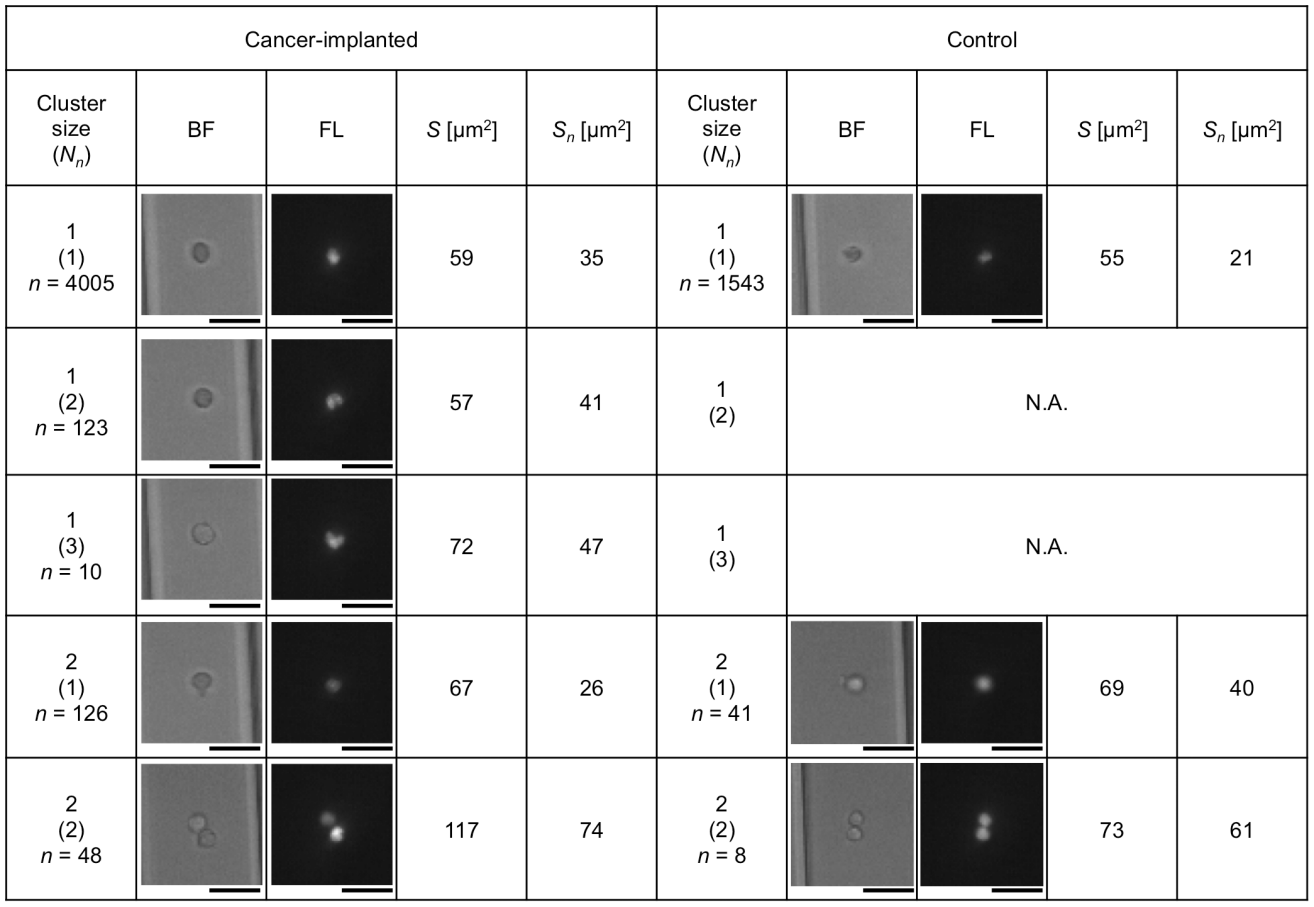
Typical cell images for single and double cells in cancer cell-implanted and control blood. Each data count (*n*) indicates the image number having the same cluster size and *N_n_*. Bars, 20 µm.

Obtained pictures were manually analyzed one by one with measured values of *S* and *S_n_*. Figure 7 shows examples of single- and double-cell images having one, two, or three nuclei obtained from cancer-implanted and control blood, respectively. As shown in Fig. 7, the following 3 results were obtained: (i) single cells having multiple nuclei numbering more than two were specifically included in the cancer cell-implanted blood (count of 133, 3.2% of total single cells in cancer-implanted samples), (ii) two-cell clusters having only one nucleus seemed to be single cells to which a small particle (possibly debris of a hemolyzed red cell) was attached (count of 126, 72% of total two-cell clusters in cancer-implanted samples and count of 41, 84% of total two-cell clusters in control), and (iii) two-cell clusters having two nuclei were either true clusters or two independent cells flowing alongside each other (count of 48, including 2 clusters having 3 nuclei caused by the inclusion of a cell with multiple nuclei, 28% of total two-cell clusters in cancer-implanted samples and count of 8, 16% of total two-cell clusters in control). The first of these results shows the potential for the detection of implanted cancer cells having multiple nuclei, and the second can be thought of as single cells in general. The third in principle makes it difficult to distinguish two-cell clusters from two single cells using pictures; therefore, such two-cell “clusters” were also contained in control blood.

**Figure 8:**
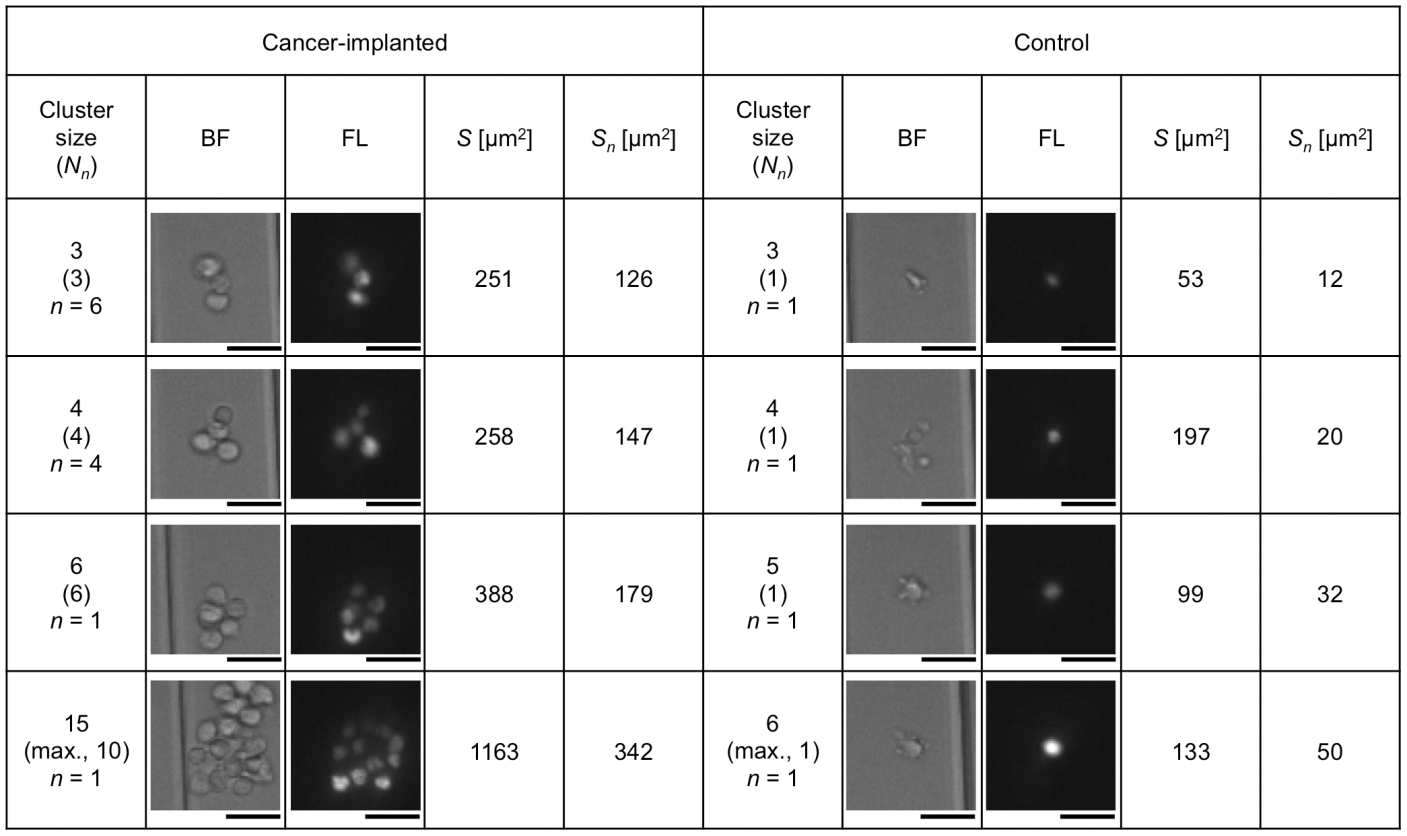
Typical cell images for clustered cells composed of more than three cells in cancer cell-implanted and control blood. Each data count (*n*) indicates the image number having the same cluster size and *N_n_*. Bars, 20 µm.

**Figure 9:**
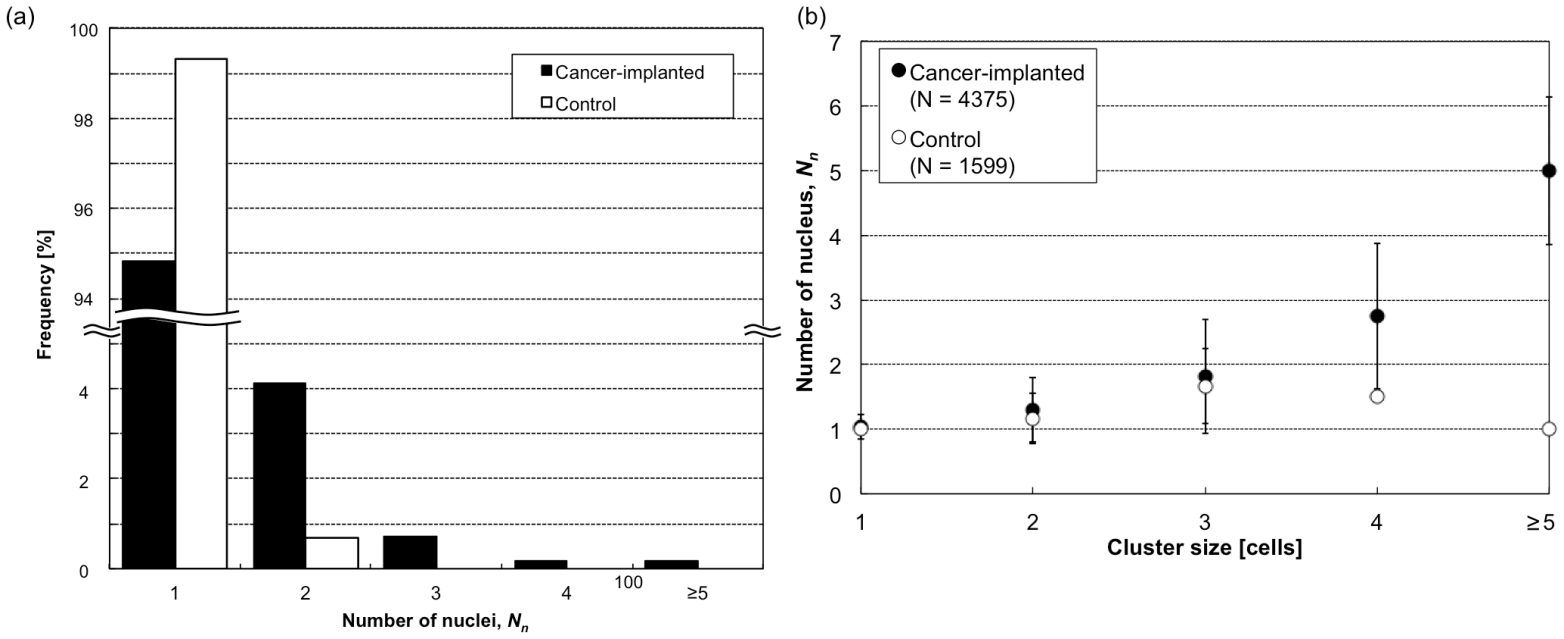
Summary of the number of nuclei, *N_n_*. (a) A histogram of *N_n_* obtained from cancer cell-implanted and control blood. (b) The relationship between *N_n_* and cell cluster size.

Figure 8 shows typical clustered cells composed of more than 3 cells. As shown in the figure and also in Table 1, a few clusters composed of more than 3 cells were also detected in control blood (count of 7 in total), with the maximum cell number of 6. However, they seemed to be single or two independent cells to which small particles were attached (i.e., the same as result (ii) in Fig. 7), which could also be confirmed by the number of nuclei, *N_n_*, in the cluster, which had a maximum of 2. On the other hand, clusters contained in cancer-implanted blood were composed of more than 3 cells, with 15 cells at maximum, which was also confirmed by *N_n_* in the cluster being more than 3. It is unlikely for more than 3 cells to be flowing alongside each other; therefore, we concluded that clusters composed of more than 3 cells containing more than 3 nuclei were truly clustered cells in the blood. Such large clusters were contained in cancer cell-implanted blood at a count of 33 (7 counts, 21% of 3-cell clusters, 12 counts, 75% of 4-cell clusters, 14 counts, 100% of ≥5-cell clusters, and 0.8% of the total). Measured values of *N_n_* are summarized in Fig. 9 (a) (and also in Table 1). As shown in this figure, more than 99% of images in control blood had a single nucleus, and cell clusters having more than 3 nuclei were not contained in the blood. Figure 9 (b) also shows *N_n_* summarized from the perspective of cluster size. As shown in the figure, large clusters in cancer-implanted blood had many nuclei, typically more than 3, indicating the possibility of the cluster formation of CTCs in the blood.

**Figure 10:**
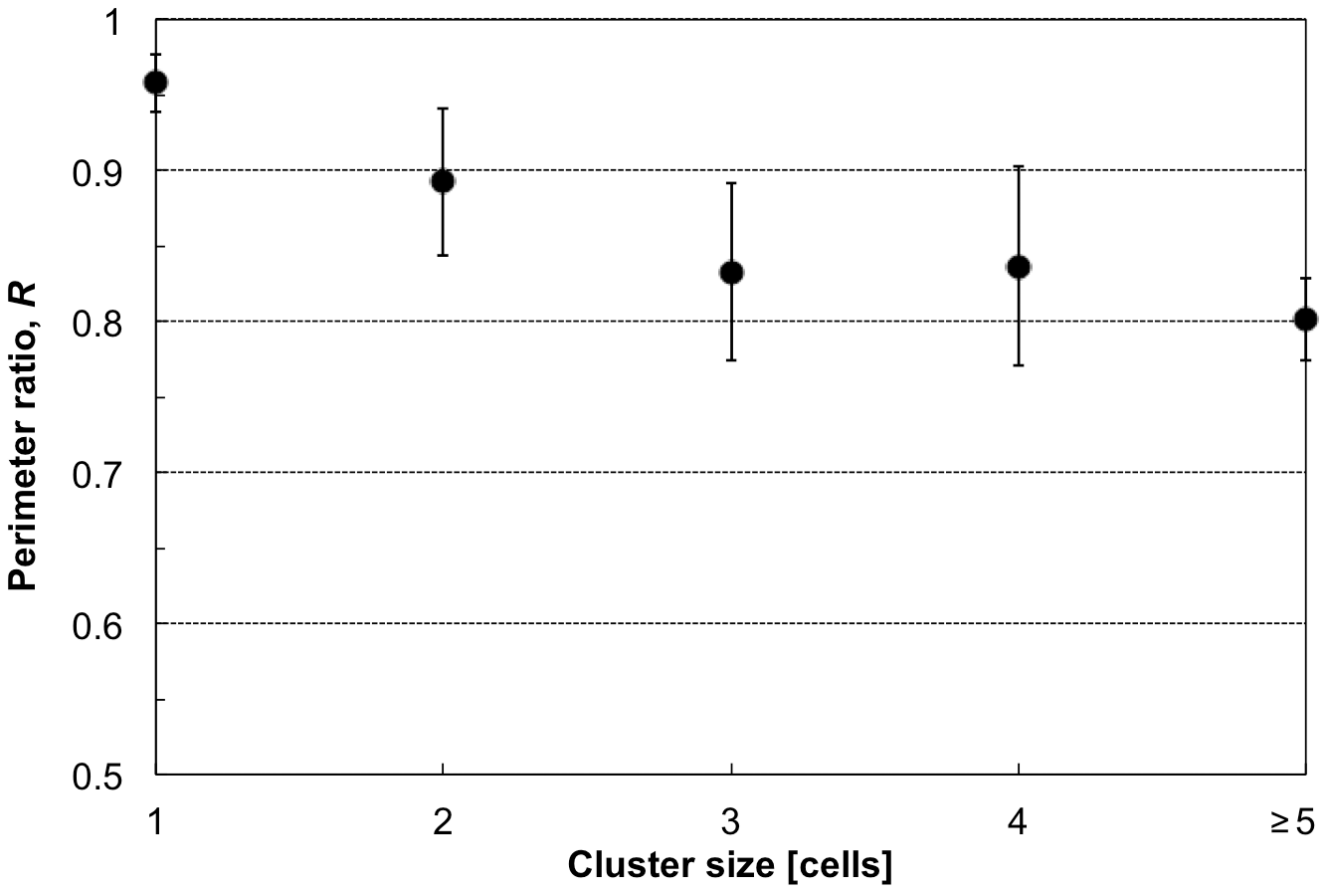
The relationship between perimeter ratio, R, and cell cluster size obtained from cancer cell-implanted blood.

As shown in the above results, *N_n_* is one useful imaging biomarker to identify cell clusters in blood; however, only using this marker for identification is insufficient because single cells having multiple nuclei were also contained in cancer cell-implanted blood, as shown in Fig. 7; therefore, we evaluated another imaging biomarker, perimeter ratio (*R*), for the identification of clustered cells. *R* is defined as the ratio between the actual perimeter obtained from the cell image and the perimeter calculated with a circle approximation of *S*. A low value of *R* indicates distorted conformation of the cell away from a circular shape, which was expected for cell clusters. Figure 10 shows the relationship between the average value of *R* and cell cluster size for a cancer-implanted sample (detailed numbers are also shown in Table 1). As shown in the figure and table, all single cells had *R* higher than 0.90, with an average of 0.96, indicating that all cells having *R* smaller than 0.90 were clusters composed of more than 2 cells. On the other hand, *R* values for clusters composed of more than 2 cells were lower than 0.90 on average, and in detail, 131 clusters in cancer-implanted samples (55% of all clusters) and 55 clusters in control (98% of all clusters) had *R* lower than 0.90. Moreover, all large clusters composed of more than 3 cells having more than 3 nuclei, specifically observed only in cancer-implanted blood, had *R* lower than 0.90. These results indicate that more than half of the clusters, especially large clusters, could be identified by using *R* as an imaging biomarker.

**Figure 11:**
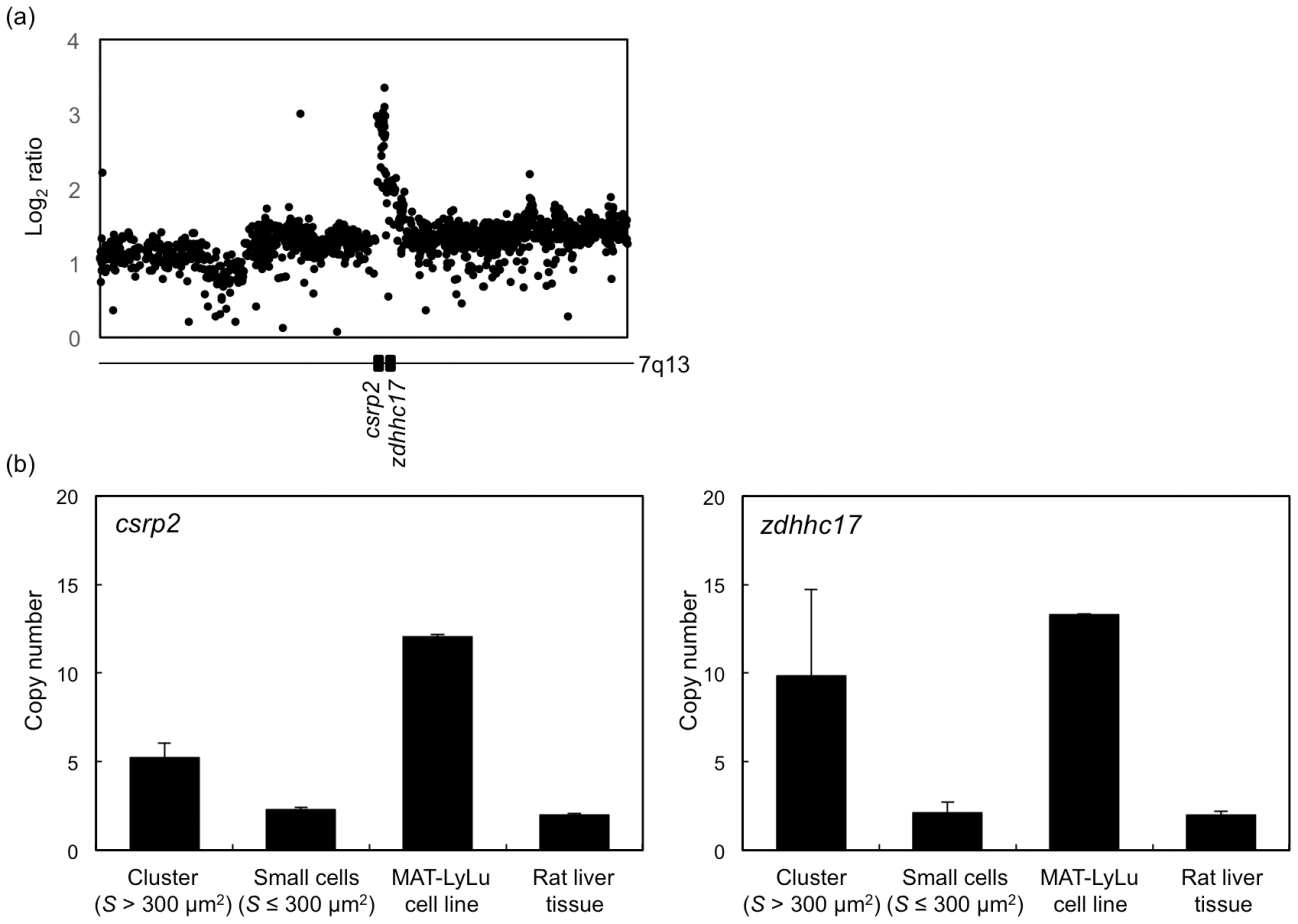
Results of quantitative gene copy number assays. (a) Results of CGH assays performed for the MAT-LyLu cell line. Liver tissue of the rat was used as a reference. Gene amplifications for *csrp2* and *zdhhc17* located on chromosome 7q13 were found. (b) Results of TaqMan copy number assays performed with clusters larger than 300 µm^2^ collected in the collection reservoir, and cells smaller than 300 µm^2^ collected in the discarded reservoir. Results of the assays for the MAT-LyLu cell line (positive control) and liver tissue (negative control) are also shown.

According to the above results, large cluster formation of cancer cells in the blood was strongly expected. To confirm this, clusters larger than 300 µm^2^ were collected by performing cell sorting in the chip, and their cell types were identified by measuring genome errors in the cells. Firstly, target genes that were included in the MAT-LyLu chromosome with abnormal copy numbers were searched by comparative genomic hybridization (CGH) assay using the cell line, with liver tissue of the rat as a reference. Two particularly abundant genes, *csrp2* and *zdhhc17* located on chromosome 7q13, were found (Fig. 11 (a)) and set as target genes for the identification of cancer cells in the blood. Next, the TaqMan copy number assay was performed for cells collected in both the collection reservoir and the discarded reservoir (see Fig. 3). From the results, increases of copy numbers for both *csrp2* and *zdhhc17* were specifically observed for clustered cells collected in the collection reservoir (Fig. 11 (b)). These results indicate that large clusters, which were specifically observed in cancer cell-implanted blood, were CTCs.

## Discussion

In this study, four imaging biomarkers, cell area, nucleus area, number of nuclei, and perimeter ratio (*S*, *S_n_*, *N_n_*, and *R*),were evaluated for the identification of cell clusters in the blood. From the results, some threshold values were obtained for each imaging biomarker, namely, (1) *S* larger than 200 µm^2^ and (2) *S_n_* larger than 90 µm^2^ were specific to cancer cell-implanted blood. In addition, (3) *N_n_* higher than 3 was also specific to cancer cell-implanted blood. Finally, (4) all clustered cells composed of more than 3 cells having *N_n_* higher than 3, which was specific to cancer cell-implanted blood, had *R* lower than 0.90. According to these results, the use of *R* is one useful approach for the identification of clustered cells having multiple nuclei numbering more than 3, which are specific to cancer cell-implanted blood. *S* and *S_n_* are also useful parameters for the identification of extremely large clusters, which are quite likely to be CTCs. For small clusters composed of two cells, it is in principle difficult to distinguish whether the cluster is an actual cluster or two independent cells flowing alongside each other by using image-based analysis. One potential approach to distinguish these possibilities is the combination of the image-based analysis suggested in this study with a molecular analytical approach, such as quantitative gene copy number assays of the targeted cells. The system developed in this study has been combined with a cell sorting unit and can perform the combination measurement of multi-imaging analysis with molecular analysis, as shown in Fig. 11, which indicates the advantage of our developed system.

For the detection of CTCs, some methods were suggested. The principles were in general separated into two kinds; one was based on the chemical reaction and the other was physical detection. The former is in general based on the labeling of target molecules on the CTCs with antibodies, and it was sometimes combined with microfabrication technologies to improve detection sensitivities [6], [7]. However, this approach sometimes yielded false-negative detection because of the variety of molecular expression levels in CTCs. For this latter case, various physical parameters of CTCs such as cell diameter [4], [16], [17] and dielectrophoretic properties [5] have been used with a combination of microfabrication technologies. According to the results in this study, cell size (*S*) is one useful parameter to find irregular cells in blood samples such as clustered cells; however, the use of only one parameter is insufficient for the exhaustive detection of CTCs. Our developed system can use various parameters including both chemical and physical properties to find target cells, which would also be useful for the detection of various CTCs.

In this study, large clusters were specifically observed in cancer cell-implanted blood, and an approach for finding these clusters in the blood has possibility for the development of a new cancer metastasis diagnostic method. Results in this study were obtained using hemolyzed blood samples *in vitro*; therefore, the large cluster formations should also be confirmed for blood *in vivo* as a next step to achieve such a new diagnostic method. One possibility for the mechanism of large cluster formation is an aggregation of implanted cancer cells by immune reaction of the rat with antibody formation. In this study, blood samples were picked up from the rat 2 weeks after implantation; therefore, time-course measurements of cluster formations after implantation might be one useful way to confirm the above possibility, and our developed system can also be used to confirm this.

## Conclusion

In this study, an on-chip multi-imaging flow cytometry system was developed to find cell clusters in blood samples. The system can take both BF and FL pictures simultaneously, and can obtain imaging biomarkers; cell area, nucleus area, number of nuclei, and perimeter ratio (*S*, *S_n_*, *N_n_*, and *R*), in real time. By using the developed system, sample blood of rats in which cancer cells had been pre-implanted was measured and compared with that of healthy rats. In terms of the results, clustered cells having (1) *S* larger than 200 µm^2^ and (2) *S_n_* larger than 90 µm^2^ were specifically observed in cancer cell-implanted blood, but were not observed in healthy rats. In addition, (3) *N_n_* higher than 3 was specific for cancer-implanted blood and (4) *R* smaller than 0.90 was specific for all clusters having *N_n_* higher than 3, which were specific for cancer-implanted blood. Finally, quantitative gene copy number assay was performed for the large clusters, and they were shown to be CTCs. These results indicate the usefulness of the imaging biomarkers for characterizing clusters, and that the developed system is useful to identify clustered CTCs in blood.
